# Expression of apoptosis-regulating genes in the rat prostate following botulinum toxin type a injection

**DOI:** 10.1186/1471-2490-12-1

**Published:** 2012-01-04

**Authors:** Tiago Gorgal, Ana Charrua, João F Silva, António Avelino, Paulo Dinis, Francisco Cruz

**Affiliations:** 1 Department of Urology, Hospital de São João, Alameda Professor Hernâni Monteiro, 4200-319, Porto- Portugal; 2 Institute for Molecular and Cell Biology (IBMC), Rua do Campo Alegre, 823, 4150-180 Porto - Portugal; 3 Department of Experimental Biology, Faculty of Medicine-University of Porto, Alameda Professor Hernâni Monteiro, 4200-319, Porto- Portugal; 4 Faculty of Nutrition and Food Sciences, Alameda Professor Hernâni Monteiro, 4200-319, Porto- Portugal; 5 Faculty of Medicine-University of Porto, Alameda Professor Hernâni Monteiro, 4200-319, Porto- Portugal

**Keywords:** Botulinum toxin, prostate, apoptosis

## Abstract

**Background:**

Onabotulinumtoxin A (OnabotA) injection has been investigated as a novel treatment for benign prostatic enlargement caused by benign prostatic hyperplasia. An OnabotA - induced volume reduction caused by sympathetic fibers impairment has been proposed as a potential mechanism of action. Our aim was to investigate the expression of apoptosis-regulating proteins in the rat prostate following OnabotA intraprostatic injection.

**Methods:**

Adult Wistar rats were injected in the ventral lobes of the prostate with 10 U of OnabotA or saline. A set of OnabotA-injected animals was further treated with 0.5 mg/kg of phenylephrine (PHE) subcutaneously daily. All animals were sacrificed after 1 week and had their prostates harvested. Immunohistochemical staining was performed for Bax, Bcl-xL and caspase-3 proteins and visualized by the avidin-biotin method. The optical density of the glandular cells was also determined, with measurement of differences between average optical densities for each group.

**Results:**

Saline-treated animals showed intense epithelial staining for Bcl-xL and a faint labelling for both Bax and Caspase-3. OnabotA-treated rats showed a reduced epithelial staining of Bcl-xL and a consistently increased Bax and Caspase-3 staining when compared with saline-treated animals. PHE-treated animals showed a stronger Bcl-xL staining and reduced staining of both Bax and Caspase-3 when compared to the OnabotA group. Mean signal intensity measurements for each immunoreaction confirmed a significant decrease of the signal intensity for Bcl-xL and a significant increase of the signal intensity for Bax and Caspase 3 in OnabotA-injected animals when compared with the control group. In OnabotA+PHE treated animals mean signal intensity for Bcl-xL, Bax and Caspase 3 immunoreactions was identical to that of the control animals.

**Conclusions:**

These results support the hypothesis that OnabotA activates apoptotic pathways in the rat prostate through a mechanism that involves sympathetic outflow impairment.

## Background

Botulinum toxin type A (BoNT/A) is one of seven serotypes of a neurotoxin produced by *Clostridium botulinum*. The toxin binds synaptic vesicle type 2 (SV2) expressed on the neuronal surface at points where synaptic vesicles fuse with the cytoplasmatic membrane. When synaptic vesicles are recycled, the neurotoxin, attached to SV2, is internalized and later cleaved into a light and heavy chain. The former is responsible for the inactivation of SNAP25 (synaptosome-associated protein of 25 kDa), a membrane protein which is crucial for the fusion of the synaptic vesicles with the neuronal membrane and subsequent release of neurotransmitters [1]. Despite its high toxicity it has several clinical applications, being approved by the US Food and Drug Administration (FDA) for treatment of blepharospasm, strabism, striated muscle dystonias and primary axillary hyperhidrosis. In addition, BoNT/A is being investigated off-label in a multitude of other diseases, including benign prostatic enlargement (BPE) caused by benign prostatic hyperplasia (BPH).

BoNT/A has been shown to consistently reduce the volume of the prostate in the rat [2] and in the dog [3]. A 30 to 50% reduction in prostate volume was also observed in several human trials in which BoNT/A was injected in moderate-to-large volume glands [4,5], although in a large phase 2 clinical trial, neither the prostate volume nor the serum PSA showed any significant decrease following the neurotoxin injection [6]. Nevertheless, it has been repeatedly demonstrated that BoNT/A induces apoptosis in rat and dog prostates, as shown by a positive deoxynucleotidyl transferase biotin-dUTP nick-end labelling (TUNEL) [2,3,7,8]. A positive TUNEL staining was also reported in one study which used prostate samples obtained from two patients following BoNT/A administration [9]. Interestingly, subcutaneous injection of the sympathicomimetic agent phenylephrine (PHE) in BoNT/A-injected rats seems to attenuate the TUNEL reaction [8], suggesting that the sympathetic nervous system could play an important role in the mechanisms of cell survival in the prostate gland, as previously forwarded by McVary and colleagues [10].

TUNEL reaction specifically labels nuclei following DNA fragmentation [11]. However, the activation of the apoptotic cascade in the prostate preceding DNA fragmentation was never investigated. Among the apoptosis regulating proteins which have been studied in the prostate are Caspase-3 and the proteins from the Bcl-2 family. The *BCL-2 *proto-oncogene family function has been postulated as one of the critical mechanisms for determining cell death, having a pivotal role in the regulation of the mitochondrial apoptosis pathway [12]. *BCL-2 *family members are divided into those that protect the cell from apoptosis, such as Bcl-2 and Bcl-xL, and those that induce apoptosis, such as Bax [12,13]. Bcl-2 and Bcl-xL overexpression in mammalian cells results in delayed apoptosis [12-15]. Bax inhibits Bcl-2 function and allows the cell to enter the death program [12]. Caspase-3 is a member of the caspase family of cysteine proteases which is central to the cell death pathway, as the extrinsic and intrinsic cell death pathways converge to activate this protein for the final execution of apoptosis [14]. Activation of caspase-3 is primarily responsible for the cleavage of poly (adenosine diphosphate-ribose) polymerase (PARP). It has been suggested that PARP contributes to cell death by depleting the cell of nicotinamide adenine dinucleotide and adenosine triphosphate [13].

In the present study, which constitutes an extension of a previous work from our group [8], we tried to further clarify the prostate cell apoptosis mechanisms induced by OnabotA by exploring changes in the expression of apoptosis-regulating proteins in the rat prostate after local administration of the neurotoxin.

## Methods

This study is an extension from a previous work published by our group [8]. Briefly, adult male Wistar rats (Charles River Laboratories, Barcelona, Spain) weighing 300-350 g were maintained in an animal house at 22°C, 60% humidity, under a dark-light cycle of 12 hours. Onabotulinumtoxin A (OnabotA, BOTOX^®^) was obtained from Allergan (Irvine, CA, USA). Phenylephrine (PHE) was supplied by ICMA (Portugal). All the experiments were performed in accordance with the revised US National Institutes of Health (NIH) Guide for the Care and Use of Laboratory Animals and with the European Communities Council Directive (86/609/EEC). The use of animals was approved by the animal subjects review board of the University of Porto, Porto, Portugal. Furthermore, all efforts were made to minimize the number of animals used.

Rats were anaesthetised with isoflurane (5% for induction and 2% for maintenance); a lower abdominal incision was performed, and the prostate was exposed and separated from the surrounding tissue. Saline (0.2 ml, n = 6) or 10 U of OnabotA in 0.2 ml of saline (n = 18) were slowly injected in several points of the ventral lobes of the prostates using a 30-gauge needle, as previously described [8]. Care was taken to administer 0.1 ml in each lobe. Six of the rats injected with OnabotA were further treated with 0.5 mg/kg of PHE subcutaneously daily. Experimental time course and OnabotA and PHE doses were chosen according to preliminary data, as stated in our previous work [8].

All animals were sacrificed after 1 week. Prostates were harvested and surrounding tissues and fat were carefully removed. The entire prostates were immersion-fixed in 4% paraformaldehyde and cut in 20 μm transverse sections using a Microm cryostat.

In the present work, prostate tissue samples from each animal were immunoreacted against the active form of Caspase-3 (ab13847, Abcam, Cambridge, UK; diluted to 1:500), Bcl-xL (#2762, Cell Signaling Technology; diluted to 1:100) and Bax (ab6943, Abcam, Cambridge, UK; diluted to 1:1000), all raised in rabbit. Immunoreactions were performed on batches for each antigen. After thawing, sections were washed and inhibition of endogenous peroxidase was obtained by incubation with 10 ml of 50% ethanol containing 0.3% H2O2. Sections were incubated with sodium borohydrate 1% for 30 m, blocked and further incubated in primary antibodies for 48 h at 4°C (Caspase-3, Bcl-xL and Bax). Sections were then incubated with a swine anti-rabbit biotinylated secondary antibody and avidin-biotin-HRP complex (ABC-Vector Laboratories, Burlingame, California). The reaction was revealed using diaminobenzidine (DAB). An omission of the primary antibody was used as negative control.

The optical density of the cytoplasm of the glandular cells following immunohistochemical reactions to Caspase-3, Bcl-xL and Bax was determined in controls and treated animals on an arbitrary scale between 0 (white pixels) and 255 (black pixels), with the Image J 1.38× software (NIH, USA) using 6 random images taken by a Zeiss microscope attached to a Zeiss Axiocam MRc5 camera. Then, the average optical density of each gland was calculated and differences between groups were analyzed using Kruskal-Wallis test followed by Dunn's Multiple Comparisons Test (GraphPad InStat, version 3.00).

## Results

**Figure 1:**
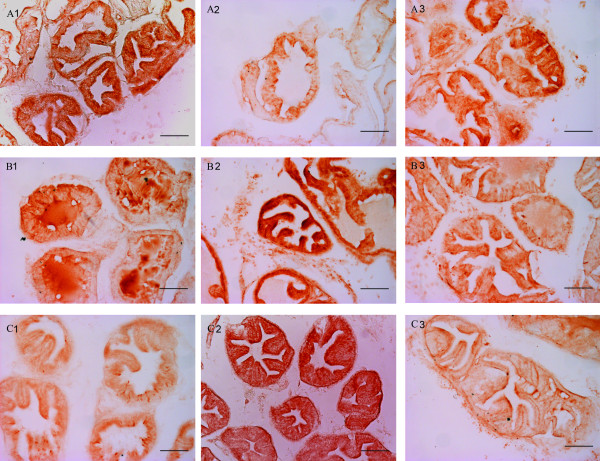
**immunohistochemical expression of Bcl-xL (A), Caspase-3 (B) and Bax (C) in glands with tall epithelial lining in the control group (1), OnaBotA group (2) and OnaBotA+PHE group (3)**. Scale bar indicates 100 μm.

**Figure 2:**
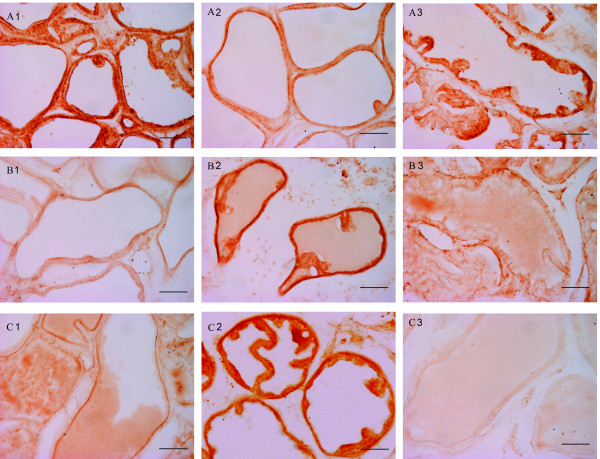
**immunohistochemical expression of Bcl-xL (A), Caspase-3 (B) and Bax (C) in glands with short epithelial lining in the control group (1), OnaBotA group (2) and OnaBotA + PHE group (3)**. Scale bar indicates 100 μm.

Saline-injected animals showed intense epithelial staining for Bcl-xL (A1) and a faint labelling for both Caspase-3 (B1) and Bax (C1), both in glands with tall (Figure 1) and short epithelial lining (Figure 2).

OnabotA-injected rats showed a reduced epithelial staining of Bcl-xL (A2) when compared with saline-treated animals in tall and short epithelial cells. The same animals showed a consistently increased Caspase-3 (B2) and Bax (C2) staining of epithelial cells (Figure 1 and 2). PHE-treated animals showed a stronger Bcl-xL (A3) staining and reduced staining of both Caspase-3 (B3) and Bax (C3), when compared to the OnabotA group (Figure 1 and 2).

**Figure 3:**
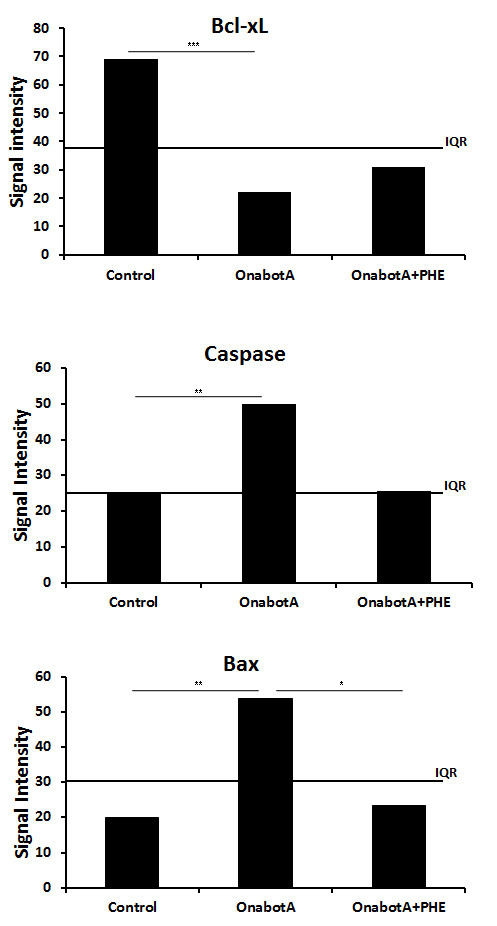
mean signal intensity measurements for Bcl-xL, Caspase 3 and Bax in control, OnabotA and OnabotA + PHE groups (* indicates p < 0.05; ** indicates p < 0.01; *** indicates p < 0.001).

Mean signal intensity measurements for each immunoreaction confirmed a significant decrease of the signal intensity for Bcl-xL and a significant increase of the signal intensity of Caspase 3 and Bax immunoreactions in OnabotA-injected animals when compared with control groups. In OnabotA+PHE treated animals, mean signal intensity for Bcl-xL, Caspase 3 and Bax immunoreactions was identical to that of control groups (Figure 3).

## Discussion

Our previous study [8], in accordance with other works [2,3,7,9], had shown the occurrence of prostate apoptosis following OnabotA. As previously mentioned, in rats injected with the toxin, prostate weight significantly decreased and there was a positive TUNEL reaction in all animals [8].

The present study further confirms the activation of the apoptotic pathway, by showing an increased expression of proapoptotic proteins and decreased expression of antiapoptotic proteins following OnabotA injection.

BoNT/A is a nonspecific neurotoxin. In fact, BoNT/A is able to impair all types of neurons that express SV2 protein on its surface and require SNAP25 protein for synaptic vesicle fusion [16]. In the prostate, cholinergic and adrenergic nerves are the most numerous. Cholinergic nerves are found mainly around acinar structures and their outflow seems to be essential for glandular secretion [17]. Adrenergic fibers are more prevalent in the stroma [17]. Sensory fibers are scarce in the prostate tissue, being restricted to the suburethral tissue and ejaculatory ducts [18]. In our previous study, we had observed that BoNT/A-injected animals subsequently treated with a subcutaneous injection of the sympathicomimetic agent PHE exhibited a decreased number of TUNEL-positive cells when compared with the BoNT/A group. In contrast, the replacement of the cholinergic outflow after BoNT/A administration with a cholinergic agonist (betanecol) could not prevent BoNT/A-induced TUNEL reaction. Our present study also confirms the important role of the sympathetic innervation for the prostate regulation in the rat. In fact, systemic PHE administration after BoNT/A injection in the gland, which is expected to replace the normal neuronal sympathetic drive impaired by the neurotoxin, prevented the overexpression of the proapoptotic proteins Bax and Caspase-3, therefore explaining why these tissues had an absent TUNEL reaction [8].

The association between the sympathetic nervous system and prostate regulation is not new. Denervation of the rat prostate achieved by bilateral pelvic ganglion denervation was shown to cause the loss of structural and functional integrity, with lower prostatic weight, decreased cell height, and reduced secretory activity [19]. Moreover, ipsilateral decreases in ventral prostate weight, DNA, and protein content were found after selective unilateral sympathectomy [10]. Spontaneous hypertensive rats which exhibit an increased sympathetic drive show a more exuberant glandular epithelium than controls [20]. In humans, prolonged administration of the alpha blockers doxazosin or terazosin to BPH patients was shown to induce epithelial and stromal cell apoptosis [21], although insufficient to cause a reduction in prostate volume [22]. In another human trial, an association was found between AUA symptom score, BPH impact index score, quality of life score and changes in systolic and sympathetic overactivity as determined by the tilt test [23]. Additionally, prostate transition zone volume was found to be independently associated with the plasma norepinephrine response to tilt [23]. Ullrich and colleagues found that an excess of physiological activity to standardized stressors correlate to transitional zone volume, total prostate gland volume, and more severe LUTS [24].

Similarly to studies in rodents, experiments using higher mammals have shown that neurotoxin injection caused profound changes in the prostate. In the dog prostate, the animal which bears more resemblance with the human gland, Lin and coworkers found that BoNT/A administration caused intense vacuolization in the cytoplasm of fibroblasts and smooth muscle cells [25]. In several human studies, OnabotA injection was followed by a reduction in prostate volume [3-5,9,26-29]. It should, however, be stated that such a reduction was not found in a recent large study comparing the effect of intraprostatic injection of different doses of OnabotA [6]. In accordance with this discrepancy, a PSA decrease reported by some studies was not confirmed by other groups [6,9]. Future studies are needed to clarify these discrepancies.

Apoptosis may play a central role both in the development and self-renewal of normal prostate and in the etiology of BPH. Shariat and colleagues [12] found an increase in the expression of survivin and Bcl-2, two antiapoptotic genes, in epithelial and stromal cells in BPH. Bcl-2 overexpression was detected in the majority of the BPH samples in a study by Zhang and colleagues which included 60 patients with histologically determined BPH. The authors concluded that the development of BPH may be associated with both stromal growth due to active stromal cell proliferation and epithelial growth due to reduced glandular apoptosis [14].

Whether these findings will contribute to the future application of BoNT/A for the treatment of BPH/LUTS is unknown. Clearly they offer a robust rational support for its use. Prostate volume reduction could reduce the static component of BPH-related bladder outlet obstruction in a manner similar to 5-alpha-reductase inhibitor drugs [30]. In addition, the impairment of the sympathetic system is also expected to reduce the dynamic component of BPH [3,25]. However, the clinical application of BoNT/A in BPH will be dependent upon the results coming from randomized clinical trials. Until now, the positive effects were seen in case series which are insufficient to draw any definitive conclusions.

## Conclusions

These results support the hypothesis that BoNT/A activates apoptotic pathways in the rat prostate through a mechanism that involves sympathetic outflow impairment. However, the scarcity of stromal component in the rat prostate precluded a thorough evaluation of the neurotoxin in the non-glandular tissue. Therefore, further studies need to be carried out in order to investigate the effect of the neurotoxin in the several cellular components of the prostate.

## List of Abbreviations

BoNT/A: Botulinum toxin type A; BPH: Benign prostatic hyperplasia; OnabotA: Onabotulinumtoxin A; PHE: phenylephrine; SNAP25: synaptosome-associated protein of 25 kDa; TUNEL: deoxynucleotidyl transferase biotin-dUTP nick-end labeling.

## Competing interests

Francisco Cruz is a consultant for Allergan, Recordati and Astellas. Paulo Dinis is a consultant for Allergan.

## Authors' contributions

The experimental work and data collection and compilation were performed by TG, AC and JFS. TG drafted the manuscript. AC carried out the statistical analysis. AA, AC, JFS, PD and FC supervised the experiments and the writing of the manuscript. All authors read and approved the final manuscript.

## Pre-publication history

The pre-publication history for this paper can be accessed here:

http://www.biomedcentral.com/1471-2490/12/1/prepub

## References

[B1] Chuang YC Kuo HC Chancellor MB Botulinum toxin for the lower urinary tract BJU Int 2010 105 1046 1058 10.1111/j.1464-410X.2010.09317.x 22299133

[B2] Doggweiler R Zermann DH Ishigooka M Schmidt RA Botox-induced prostatic involution Prostate 1998 37 44 50 10.1002/(SICI)1097-0045(19980915)37:1<44::AID-PROS7>3.0.CO;2-8 9721068

[B3] Chuang YC Tu CH Huang CC Lin HJ Chiang PH Yoshimura N Chancellor MB Intraprostatic injection of botulinum toxin type-A relieves bladder outlet obstruction in human and induces prostate apoptosis in dogs BMC Urol 2006 18 6 12 10.1186/1471-2490-6-12 PMC1481565 16620393

[B4] Maria G Brisinda G Civello IM Bentivoglio AR Sganga G Albanese A Relief of botulinum toxin of voiding disfunction due to benign prostatic hyperplasia: results of randomized, placebo-controlled study Urology 2003 62 259 264 10.1016/S0090-4295(03)00477-1 12893330

[B5] Silva J Silva C Saraiva L Silva A Pinto R Dinis P Cruz F Intraprostatic botulinum toxin type A injection in patients unfit for surgery presenting with refractory urinary retention and benign prostatic enlargement. Effect on prostate volume and micturition resumption Eur Urol 2008 53 153 159 10.1016/j.eururo.2007.08.050 17825981

[B6] McVary K Crawford ED Donnell R Drews K Kaplan S Kusek J Roehrborn C Bruskewitz R Mist 2: baseline PSA and total prostate volume predicts clinical response to intraprostatic injection of botulinum toxin for the management of LUTS J Urol 2010 183 Supp 1 690

[B7] Chuang YC Huang CC Kang HY Chiang PH Demiguel F Yoshimura N Chancellor MB Novel action of botulinum toxin on the stromal and epithelial components of the prostate gland J Urol 2006 175 1158 1163 10.1016/S0022-5347(05)00318-6 16469644

[B8] Silva J Pinto R Carvalho T Coelho A Avelino A Dinis P Cruz F Mechanisms of prostate atrophy after glandular botulinum neurotoxin type A injection: an experimental study in the rat Eur Urol 2009 56 134 140 10.1016/j.eururo.2008.07.003 18649990

[B9] Chuang YC Chiang PH Huang CC Yoshimura N Chancellor MB Botulinum toxin type A improves benign prostatic hyperplasia symptoms in patients with small prostates Urology 2005 66 775 779 10.1016/j.urology.2005.04.029 16230137

[B10] McVary KT Razzaq A Lee C Venegas MF Rademaker A McKenna KE Growth of the rat prostate is facilitated by the autonomic nervous system Biol Reprod 1994 51 99 107 10.1095/biolreprod51.1.99 7918880

[B11] Gavrieli Y Sherman Y Ben-Sasson SA Identification of programmed cell death in situ via specific labeling of nuclear DNA fragmentation J Cell Biol 1992 119 493 501 10.1083/jcb.119.3.493 1400587 PMC2289665

[B12] Shariat SF Ashfaq R Roehrborn CG Slawin KM Lotan Y Expression of survivin and apoptotic biomarkers in benign prostatic hyperplasia Urology 2005 174 2046 2050 10.1097/01.ju.0000176459.79180.d1 16217391

[B13] Vela-Navarrete R Escribano-Burgos M Farré AL García-Cardoso J Manzarbeitia F Carrasco C Serenoa repens treatment modifies Bax/Bcl-2 index expression and caspase-3 activity in prostatic tissue from patients with benign prostatic hyperplasia Urology 2005 173 507 510 10.1097/01.ju.0000150533.94952.25 15643230

[B14] Zhang X Zhang Q Zhang Z Na Yanqun Guo Y Apoptosis profiles in benign prostatic hyperplasia: close associations of cell kinetics with percent area density of histologic composition Urology 2006 68 905 910 10.1016/j.urology.2006.05.013 17070390

[B15] Boise LH González-García M Postema CE Ding L Lindsten T Turka LA Mao X Nuñez G Thompson CB Bcl-x, a bcl-2-related gene that functions as a dominant regulator of apoptotic cell death Cell 1993 74 597 608 10.1016/0092-8674(93)90508-N 8358789

[B16] Charrua A Avelino A Cruz F Modulation of urinary bladder innervation: TRPV1 and botulinum toxin A Handb Exp Pharmacol 2011 202 345 374 10.1007/978-3-642-16499-6_17 21290235

[B17] Pennefather JN Lau WA Mitchelson F Ventura S The autonomic and sensory innervation of the smooth muscle of the prostate gland: a review of pharmacological and histological studies Auton Pharmacol 2000 20 193 206 10.1046/j.1365-2680.2000.00195.x 11260358

[B18] Dinis P Charrua A Avelino A Nagy I Quintas J Ribau U Cruz F The distribution of sensory fibers immunoreactive for the TRPV1 (capsaicin) receptor in the human prostate Eur Urol 2005 48 162 167 10.1016/j.eururo.2005.01.009 15967267

[B19] Wang JM McKenna KE McVary KT Lee C Requirement of innervation for maintenance of structural and functional integrity in the rat prostate Biol Reprod 1991 44 1171 1176 10.1095/biolreprod44.6.1171 1873391

[B20] Matityahou A Rosenzweig N Golomb E Rapid proliferation of prostatic epithelial cells in spontaneously hypertensive rats: a model of spontaneous hypertension and prostate hyperplasia J Androl 2003 24 263 269 12634314 10.1002/j.1939-4640.2003.tb02671.x

[B21] Anglin IE Glassman DT Kyprianou N Induction of prostate apoptosis by alpha1-adrenoceptor antagonists: mechanistic significance of the quinazoline component Prostate Cancer Prostatic Dis 2002 5 88 95 10.1038/sj.pcan.4500561 12496995

[B22] McConnell JD Roehrborn CG Bautista OM Andriole GL Jr Dixon CM Kusek JW Lepor H McVary KT Nyberg LM Jr Clarke HS Crawford ED Diokno A Foley JP Foster HE Jacobs SC Kaplan SA Kreder KJ Lieber MM Lucia MS Miller GJ Menon M Milam DF Ramsdell JW Schenkman NS Slawin KM Smith JA Medical Therapy of Prostatic Symptoms (MTOPS) Research Group The long-term effect of doxazosin, finasteride, and combination therapy on the clinical progression of benign prostatic hyperplasia N Engl J Med 2003 349 2387 2398 10.1056/NEJMoa030656 14681504

[B23] McVary KT Rademaker A Granville LL Gann P Autonomic nervous system overactivity in men with lower urinary tract symptoms secondary to benign prostatic hyperplasia Urology 2005 174 1327 1333 10.1097/01.ju.0000173072.73702.64 16145413

[B24] Ullrich PM Lutgendorf SK Kreder KJ Physiologic reactivity to a laboratory stress task among men with benign prostatic hyperplasia Urology 2007 70 487 491 10.1016/j.urology.2007.04.048 17905102 PMC2084069

[B25] Lin AT Yang AH Chen KK Effects of botulinum toxin A on the contractile function of dog prostate Eur Urol 2007 52 582 589 10.1016/j.eururo.2007.03.002 17386969

[B26] Brisinda G Cadeddu F Vanella S Mazzeo P Marniga G Maria G Relief by Botulinum Toxin of Lower Urinary Tract Symptoms Owing to Benign Prostatic Hyperplasia: Early and Long-Term Results Urology 2009 73 90 94 10.1016/j.urology.2008.08.475 18995889

[B27] Silva J Pinto R Carvalho T Botelho F Silva P Oliveira R Silva C Cruz F Dinis P Intraprostatic Botulinum Toxin Type A injection in patients with benign prostatic enlargement: duration of the effect of a single treatment BMC Urol 2009 9 9 10.1186/1471-2490-9-9 19682392 PMC2734751

[B28] Kuo HC Prostate botulinum A toxin injection- an alternative treatment for benign prostatic obstruction in poor surgical candidates Urology 2005 65 670 674 10.1016/j.urology.2004.10.077 15833506

[B29] Chuang YC Chiang PH Yoshimura N De Miguel F Chancellor MB Sustained beneficial effects of intraprostatic botulinum toxin type A on lower urinary tract symptoms and quality of life in men with benign prostatic hyperplasia BJU Int 2006 98 1033 1037 10.1111/j.1464-410X.2006.06479.x 16956361

[B30] Roehrborn CG Siami P Barkin J Damião R Major-Walker K Nandy I Morrill BB Gagnier RP Montorsi F The effects of combination therapy with dutasteride and tamsulosin on clinical outcomes in men with symptomatic benign prostatic hyperplasia: 4-year results from the CombAT study Eur Urol 2010 57 123 131 10.1016/j.eururo.2009.09.035 19825505

